# Three-Item Dimensions of Anger Reactions Scale

**DOI:** 10.1001/jamanetworkopen.2023.54741

**Published:** 2024-02-05

**Authors:** David Forbes, Cynthia A. LeardMann, Ellie Lawrence-Wood, Javier Villalobos, Kelsey Madden, Ian A. Gutierrez, Sean Cowlishaw, Jenelle Baur, Amy B. Adler

**Affiliations:** 1 Phoenix Australia–Centre for Posttraumatic Mental Health, Department of Psychiatry, University of Melbourne, Carlton, Australia; 2 Deployment Health Research Department, Naval Health Research Center, San Diego, California; 3 Leidos, San Diego, California; 4 Center for Military Psychiatry and Neuroscience, Walter Reed Army Institute of Research, Silver Spring, Maryland

## Abstract

**Importance:**

Problematic anger is prevalent and associated with adjustment difficulties in military populations. To facilitate measurement of problematic anger, a very brief valid measure is needed.

**Objective:**

To reduce the Dimensions of Anger Reactions 5-item (DAR-5) scale to a very brief measure.

**Design, Setting, and Participants:**

This cross-sectional study used survey data collected between 2014 and 2016 in the Australian Transition and Well-Being Research Programme and US Millennium Cohort Study. Participants were service members who were actively serving or had transitioned out of the military (separated). Statistical analyses were performed from September 2021 to June 2023.

**Main Outcomes and Measures:**

The DAR-5 was reduced to the 3 experiential items: frequency, intensity, and duration (the DAR-3). Psychometrics for the DAR-3 and DAR-5 were compared in terms of standardized Cronbach α, positive screening result, mean, and SD. Analyses were stratified by Australian and US military service status cohorts (active duty and separated).

**Results:**

A total of 71 010 participants were included from Australia and the US. Of 10 900 Australian participants (8145 active duty participants [74.7%]; 2755 separated participants [25.3%]), 5893 (55.2%) were aged 40 years or older and 8774 (80.5%) were male; of 60 110 US participants (24 706 active duty participants [41.1%]; 35 404 separated participants [58.9%]), 28 804 (47.9%) were aged 30 to 39 years and 43 475 (72.3%) were male. The DAR-3 demonstrated good internal consistency in the active duty (Australia: mean [SD] score, 4.97 [2.5]; α = 0.90; US: mean [SD] score, 5.04 [2.6]; α = 0.87) and separated (Australia: mean [SD] score, 6.53 [3.4]; α = 0.92; US: mean [SD] score, 6.05 [3.2]; α = 0.91) samples. The cutoff score of 8 or greater on the DAR-3 had optimal sensitivity and specificity across all samples. DAR-3 and DAR-5 were associated with posttraumatic stress disorder (PTSD), depression, aggression, and relationship conflict. While the scales did not significantly differ in their associations with PTSD, depression, and relationship conflict, the magnitude of association for aggression was significantly lower in US samples using the DAR-3 (eg, US active duty sample: DAR-5 OR, 9.96; 95% CI, 9.01-11.00; DAR-3 OR, 8.36; 95% CI, 7.58-9.22).

**Conclusions and Relevance:**

In this cross-sectional study of a very brief measure of anger, each item contributed to the overall strength of the measure without losing psychometric strength compared with the DAR-5. The consistency of these findings across military and veteran samples in Australian and US populations demonstrated the psychometric robustness of the DAR-3.

## Key Points

**Question:**

Can the 5-item Dimensions of Anger Reaction (DAR-5) scale be shortened to produce a 3-item measure of problematic anger (DAR-3) that retains the DAR-5’s psychometric properties?

**Findings:**

In this cross-sectional study using survey data from 71 010 participants from 2 large military samples in Australia and the US, the DAR-3 exhibited robust psychometric properties.

**Meaning:**

These findings suggest that DAR-3 offers a very brief, valid, and effective measure of problematic anger to reduce participant burden on surveys and offers a cutoff score that supports its practical utility across military and veteran populations.

## Teaser

This cross-sectional study uses data from 2 large Australian and US military cohorts to reduce the Dimensions of Anger Reactions 5-item scale (DAR-5) to a briefer 3-item measure.

## Introduction

Estimates suggest that 1 in 6 active-duty military personnel and 1 in 3 veterans exceed criteria on a measure of problematic anger.^1,2^ Not only is problematic anger prevalent, it is associated with difficulties across psychosocial and economic domains. In studies of US military personnel, problematic anger has been associated with worse psychological health and challenges with personal relationships, parenting, financial well-being, employment, and housing, even after adjusting for co-occurring mental health problems.^3^ In addition, studies of Australian military personnel have identified that problematic anger from point of recruitment has a negative impact on mental health trajectories.^4^ Moreover, analysis of Australian veteran data indicates problematic anger is associated with increased suicidality and interpersonal violence, after adjusting for demographic and mental health variables.^2^ Problematic anger has also been associated with self-harm^5^ and aggression^6^ in US samples. These findings illustrate the risk that problematic anger poses for military personnel, separated veterans, and their families.

The standard measure of anger, the Speilberger State Trait Anger Expression Inventory,^7^ while providing a robust and thorough assessment of problematic anger, is lengthy and lacks a cutoff score. These characteristics have limited its use in studies, organizational screening, and clinical settings. The 5-item Dimensions of Anger Reactions scale (DAR-5),^8,9^ adapted from the original 7-item scale,^10^ addresses these concerns by offering a short, validated measure with an empirically developed cutoff score.

The DAR-5’s psychometric properties have been established in clinical and community samples across US,^8,11^ Australian,^9^ French,^12^ and Arabic-speaking^13^ populations. The cutoff score of 12 or greater has also been replicated in these samples, and the DAR-5 is being translated for military studies in Norway, the Netherlands, and Germany.

Despite the utility of the DAR-5, there is continuous pressure on studies to minimize participant burden, maximize response rates, and assess a variety of constructs.^14,15,16^ This pressure may drive studies to either exclude measurement of certain constructs^14^ or shorten scales without guidance from validation studies.^17^ In the case of problematic anger, the absence of a reliable and valid very brief measure may limit its inclusion in studies and clinical contexts. Therefore, the development of a very brief version of the DAR-5 is needed to support wide-scale assessment of anger in surveys and intervention-based research.

The aim of this cross-sectional study was to reduce the length of the DAR-5, producing a valid and reliable very brief measure of problematic anger. Item reduction was based on theoretical grounds and focused on presentation and severity of the subjective experience of anger rather than the result of anger. Thus, to develop the 3-item Dimensions of Anger Reactions (DAR-3) scale, we retained the first 3 DAR-5 items assessing anger frequency, intensity, and duration and discarded the 2 items assessing aggression and interference with social functioning. This approach follows previous efforts to streamline short scales (eg, the 10-item Alcohol Use Disorders Identification Test to 3 items [AUDIT-C],^18^ the 9-item Patient Health Questionnaire [PHQ] to 2 items,^19^ and the 20-item PTSD [posttraumatic stress disorder] Checklist to 4 items^15^).

To address this objective, we examined the relative psychometric properties and concurrent validity between the DAR-5 and DAR-3 in 2 large-scale military studies from Australia and the US. In addition, we sought to identify screening cutoff criteria for the 3-item measure. Testing the DAR-3 can inform the development of a streamlined measure for assessing problematic anger in a variety of military and veteran populations.

## Methods

### Population and Data Source

Study samples were drawn from 2 military population-based research studies: the Australian Transition and Well-Being Research Programme^20^ (hereafter, *the Programme*) and the US Millennium Cohort Study.^21,22^ The Programme was approved by the Defence and Department of Veterans’ Affairs Human Research Ethics Committees. The study protocol for the US Millennium Cohort Study was approved by the Naval Health Research Center institutional review board. All participants provided voluntary, informed consent. In both studies, participants completed an online or paper survey that covered topics such as military background, mental health, physical health, and health care utilization. This study followed the Standards for Reporting of Diagnostic Accuracy (STARD) reporting guideline.

### Australian Sample

The Australian data were taken from the Programme, a large-scale survey of 12 806 Australian Defence Force (ADF) service members and veterans, conducted in 2015. For these analyses, the active-duty sample consisted of ADF members who were currently serving in 2015 (8480 individuals). Of 4326 service members who had transitioned out of regular full-time service between 2010 and 2014, the separated sample included former service members with no regular service commitments (2907 individuals); ex-serving inactive reservists were excluded from all analyses (1419 individuals). After excluding participants missing data on 1 or more DAR-5 items (335 active duty individuals; 152 separated individuals), 10 900 participants were included in analyses. Previous investigations have found the Programme was broadly representative of the ADF population.^20^

### United States Sample

The US data set was taken from the Millennium Cohort Study, a population-based longitudinal study of service member and veteran health. For these analyses, participants included those who completed a full-length 2014 to 2016 survey (103 245 individuals). Of those, 26 290 individuals were active-duty service members and 36 731 individuals were former active duty service members (ie, separated). Supplementary analyses were conducted on the remaining 40 224 current and former Reserve and National Guard members. The primary analysis, which focused on current and separated active duty personnel, included 60 110 participants, after excluding those missing data on 1 or more DAR-5 items (1584 active duty individuals; 1327 separated individuals). Details regarding the Millennium Cohort study have been provided elsewhere,^21,22,23^ and investigations have found the cohort to be representative of the US Armed Forces population.^21,22^

### Measures

The DAR-5 measures anger frequency, intensity, duration, aggression, and impact on social functioning using a 5-point scale. Item scores were summed, with a possible range of 5 to 25; scores of 12 or greater indicate problematic anger.^8^

Posttraumatic stress disorder (PTSD) symptoms were measured using the 17-item PTSD Checklist civilian version (PCL-C), which aligns with PTSD criteria in the *Diagnostic and Statistical Manual of Mental Disorders* (Fourth Edition) (*DSM-IV*). Responses, rated on a 5-point scale, were summed, with a possible range of 17 to 85; a score of 30 or greater indicates probable PTSD.^24^

Depression symptoms were assessed using the 8-item PHQ (PHQ-8). Items were rated on a 4-point scale and summed, with a possible range of 0 to 24. A score of 10 or greater indicates probable depression.^25,26^

Aggression was measured with 1 item using a 5-point scale in both samples. In the Australian sample, participants reported how frequently they engaged in physical violence in the last month (ie, “get into a fight with someone and hit the person”). This item was taken from the 2010 Mental Health Prevalence and Well-Being Study.^27^ In the US sample, participants reported how frequently they engaged in physical violence in the last month (ie, “get angry with someone and kick/smash something, get into a fight, hit someone, or threaten someone with physical violence?”). This item was adapted from previous military studies.^28^ For both study samples, reporting at least 1 time indicated aggression.

Relationship conflict was measured with 1 item using a 3-point scale in both studies. In the Australian sample, relationship conflict was assessed using an item from previous studies.^29^ Participants were asked “In general, how would you describe your relationship?” with 0 indicating no tension; 1, some tension; and 2, a lot of tension. In the US sample, participants responded to a PHQ item^30^ in which they rated how bothered (range, 0-2, with 0 indicating not bothered; 1, bothered a little; and 2, bothered a lot) they were by “difficulties with husband/wife, partner/lover, or boyfriend/girlfriend.” For both study samples, scores of 1 or greater were coded as relationship conflict.

For both countries, demographics included age and sex; military characteristics included rank and service branch. For the Australian sample, demographics were collected on the survey, and military characteristics were obtained from the Defence Personnel Management Key System. For the US sample, demographics and military characteristics were obtained from the Defense Manpower Data Center; information on sex, race, and ethnicity was provided by service members using categories defined by the service branches. In this study, race and ethnicity for US participants are categorized as American Indian; Asian or Pacific Islander; Black, non-Hispanic; Hispanic; multiracial; and White, non-Hispanic. Race and ethnicity were assessed for the purpose of demographic reporting.

### Statistical Analysis

Descriptive analyses examined characteristics of the 4 samples (ie, active duty and separated for Australia and the US). Standardized Cronbach α, means, SDs, and the number of positive screening results for the DAR-5 and DAR-3 were calculated for each sample. Additionally, coefficients for α if each item were deleted were calculated for the DAR-5 and DAR-3.

Concurrent validity of the DAR-3 was assessed using sensitivity, specificity, accuracy, and Cohen κ against the DAR-5 criterion score of at least 12 for each possible score of the DAR-3 in the 4 samples. The criterion score for the DAR-3 was established through a holistic assessment of these measures of agreement by identifying the score that maximized Cohen κ without notable loss to either the sensitivity or specificity of the new measure.^31^ Following identification of an optimal criterion score for the DAR-3, assessment of concurrent validity was examined through computation of confusion matrices for the DAR-5 and DAR-3 for the 4 samples using the respective criterion scores.

Predictive validity of the DAR-3 was assessed through a comparison of odds ratios (ORs) derived from binomial logistic regression models. For the 4 samples, 2 logistic regression models—1 with the DAR-5 as the independent variable and 1 with the DAR-3 as the predictor—were conducted on each outcome: PTSD, depression, aggression, and relationship conflict. Corresponding bivariate ORs were obtained from 16 pairs of models (4 samples × 4 outcomes). ORs were compared with standardized *z* scores to determine whether there were significant differences in estimates between the DAR-5 and the DAR-3.^32^ Since a large portion of the US military consists of Reserve and National Guard members, a supplemental analysis examined the relative psychometric properties of the DAR-3 in this population (eMethods in Supplement 1). *P* values were 2-sided, and statistical significance was set at *P* = .05. Statistical analyses were conducted in the R language for statistical computing, (R Project for Statistical Computing) version 4.1.2 in the Australian sample and version 4.2.0 in the US sample from September 2021 to June 2023.

## Results

### Descriptive Statistics

A total of 71 010 participants were included in analyses. Of 10 900 Australian participants, 8145 (74.7%) were active duty and 2755 (25.3%) were separated (Table 1). Among Australian participants, 5893 (54.9%) were aged 40 years or older, 8774 (80.5%) were male, 5466 (50.1%) were noncommissioned officers, and 5131 (47.1%) were in the Army. While race and ethnicity were not measured, the racial and ethnic composition of the ADF is primarily Anglo-Celtic.^33^ Younger participants were more likely to be missing DAR-5 items.

**Table 1.** Characteristics of Australian and US Participants by Separation Status at Time of Survey Completion

Characteristic	Participants, No. (%)					
	Australia			US		
	Total (n = 10 900)	Active duty (n = 8145)	Separated (n = 2755)	Total (n = 60 110)	Active duty (n = 24 706)	Separated (n = 35 404)
Age, y						
18-29	1546 (14.2)	974 (12.0)	572 (20.8)	8617 (14.3)	4858 (19.7)	3759 (10.6)
30-39	3287 (30.2)	2470 (30.3)	817 (30.0)	28 804 (47.9)	14 646 (59.3)	14 158 (40.0)
≥40	5893 (54.1)	4558 (56.0)	1335 (48.4)	22 689 (37.7)	5202 (21.1)	17 487 (49.4)
Missing	174 (1.6)	143 (1.8)	31 (1.1)	0	0	0
Sex						
Male	8774 (80.5)	6450 (79.2)	2324 (84.4)	43 475 (72.3)	18 560 (75.1)	24 915 (70.4)
Female	2126 (19.5)	1695 (20.8)	431 (15.6)	16 635 (27.7)	6146 (24.9)	10 489 (29.6)
Race and ethnicity						
American Indian	NA	NA	NA	803 (1.3)	337 (1.4)	466 (1.3)
Asian or Pacific Islander	NA	NA	NA	2687 (4.5)	1389 (5.6)	1298 (3.7)
Black, non-Hispanic	NA	NA	NA	6333 (10.5)	2575 (10.4)	3758 (10.6)
Hispanic	NA	NA	NA	4441 (7.4)	1905 (7.7)	2536 (7.2)
Multiracial	NA	NA	NA	829 (1.4)	387 (1.6)	442 (1.3)
White, non-Hispanic	NA	NA	NA	45 006 (74.9)	18 108 (73.3)	26 898 (76.0)
Missing	NA	NA	NA	11 (<0.1)	5 (<0.1)	6 (<0.1)
Military rank^a^						
Junior enlisted	1347 (12.4)	561 (6.9)	786 (28.5)	9630 (16.0)	497 (2.0)	9133 (25.8)
NCOs or WOs	5466 (50.2)	4161 (51.1)	1305 (47.4)	33 304 (55.4)	14 706 (59.5)	18 598 (52.5)
Officers or WOs	4087 (37.5)	3423 (42.0)	664 (24.1)	17 176 (28.6)	9503 (38.5)	7673 (21.7)
Service branch						
Army	5131 (46.6)	3362 (41.3)	1768 (64.2)	22 390 (37.3)	8713 (35.3)	13 677 (38.6)
Navy	2419 (22.0)	1959 (24.1)	460 (16.7)	12 243 (20.4)	4413 (17.9)	7830 (22.1)
Air Force	3451 (31.4)	2824 (34.7)	527 (19.1)	18 193 (30.3)	8951 (36.2)	9242 (26.1)
Marine Corps	NA	NA	NA	5905 (9.8)	1755 (7.1)	4150 (11.7)
Coast Guard	NA	NA	NA	1379 (2.3)	874 (3.5)	505 (1.4)
Mental health and well-being^b^^,^^c^						
PTSD	2484 (25.0)	1377 (18.4)	1107 (44.9)	16 729 (28.3)	4332 (17.9)	12 397 (35.5)
Depression	2162 (19.9)	1188 (14.6)	974 (35.4)	10 406 (17.3)	2379 (9.6)	8027 (22.7)
Aggression	197 (1.8)	79 (1.0)	118 (4.3)	6996 (11.7)	1936 (7.9)	5060 (14.3)
Relationship conflict	3867 (46.9)	2888 (45.3)	979 (52.2)	22 563 (37.8)	8121 (33.0)	14 442 (41.1)
Problematic anger^c^						
DAR-5	1999 (18.3)	1071 (13.1)	928 (33.5)	11 519 (19.2)	3070 (12.4)	8449 (23.9)
DAR-3	2104 (19.3)	1154 (14.2)	950 (34.3)	12 874 (21.4)	3617 (14.6)	9257 (26.1)

Abbreviations: DAR-3, 3-item Dimensions of Anger Reactions; DAR-5, 5-item Dimensions of Anger Reactions; NA, not applicable; NCO, noncommissioned officer; PTSD, posttraumatic stress disorder; WO, warrant officer.

^a^ WO positions differ in the Australian and US military; thus, Australian WOs were placed with NCOs, and US WOs were placed with the officers.

^b^ Calculations were based on participants who had nonmissing data for the specific mental health or well-being scale; due to missing data, denominators ranged from 6369 to 8132 (Australia active duty) and 1877 to 2749 (Australia separated), and 24 245 to 24 690 (US active duty) and 34 960 to 35 356 (US separated).

^c^ Numbers and percentages represent participants scoring above defined cut offs (ie, PTSD, ≥30; depression, ≥10; aggression, ≥1 time; relationship conflict, ≥1; DAR-5, ≥12; DAR-3, ≥8).

Of 60 110 US participants, 24 706 (41.1%) were active duty and 35 404 (58.9%) were separated (Table 1). Among US participants, 28 804 (47.9%) were aged 30 to 39 years, 43 475 (72.3%) were male, 33 304 (55.4%) were noncommissioned officers, and 22 390 (37.2%) were in the Army. There were 803 American Indian or Alaska Native participants (1.3%); 2687 Asian or Pacific Islander participants (4.5%); 6333 Black, non-Hispanic participants (10.5%); 4441 Hispanic participants (7.4%); 829 multiracial participants (1.4%); and 45 006 White, non-Hispanic participants (74.9%). Participants missing DAR-5 items were more likely to be younger and identify as Asian or Pacific Islander; Black, non-Hispanic; or Hispanic.

### Psychometric Analysis

The DAR-5 had good internal consistency in both the active-duty (Australia: mean [SD] score, 7.66 [3.7]; α = 0.91; US: mean [SD] score, 7.63 [3.6]; α = 0.89) and separated (Australia: mean [SD] score, 10.16 [5.3]; α = 0.93; US: mean [SD] score, 9.17 [4.9]; α = 0.92) samples. In the active duty samples, 1071 Australian participants (13.1%) and 3070 US participants (12.4%) had screening results positive for problematic anger on the DAR-5; in the separated samples, 928 Australian participants (33.7%) and 8449 US participants (23.9%) participants had positive screening results.

Our selection of the anger frequency, intensity, and duration items from the DAR-5 on theoretical grounds for the DAR-3 scale was supported by our α-if-deleted analysis. Specifically, when each of the DAR-5 items was deleted, internal consistency decreased slightly (Table 2) but for the final 2 DAR-5 items (aggressive impulses and interference with social functioning), internal consistency decreased the same or less than the other 3 items across all 4 samples. The DAR-3 demonstrated comparable Cronbach α coefficients in both the active duty (Australia: mean [SD] score, 4.97 [2.5]; α = 0.90; US: mean [SD] score, 5.04 [2.6]; α = 0.87) and separated (Australia: mean [SD] score, 6.53 [3.4]; α = 0.92; US: mean [SD] score, 6.05 [3.2]; α = 0.91) samples. In the active-duty samples, 1154 Australian participants (14.2%) and 3617 US participants (14.6%) had positive screening results for problematic anger on the DAR-3. In the separated samples, 950 Australian participants (34.5%) and 9257 US participants (26.1%) had positive screening results.

**Table 2.** Internal Consistency and Psychometrics of DAR-5 and DAR-3 in Australia and the US by Separation Status

Measure^a^	Australia				US			
	Active duty (n = 8145)		Separated (n = 2755)		Active duty (n = 24 706)		Separated (n = 35 404)	
	Mean (SD)	α	Mean (SD)	α	Mean (SD)	α	Mean (SD)	α
**DAR-5 Scale**								
Full Scale	7.66 (3.7)	0.91	10.16 (5.3)	0.93	7.63 (3.6)	.89	9.17 (4.9)	0.92
Individual items^a^								
1. “I found myself getting angry at people or situations”	2.01 (1.0)	0.90	2.54 (1.1)	0.92	1.86 (1.0)	.86	2.22 (1.2)	0.90
2. “When I got angry, I got really mad”	1.54 (0.9)	0.88	2.09 (1.3)	0.90	1.71 (1.0)	.85	2.06 (1.2)	0.90
3. “When I got angry, I stayed angry”	1.43 (0.9)	0.88	1.96 (1.2)	0.91	1.47 (0.9)	.85	1.76 (1.1)	0.90
4. “When I got angry at someone, I wanted to hit them”	1.29 (0.8)	0.91	1.76 (1.2)	0.93	1.33 (0.8)	.87	1.60 (1.1)	0.91
5. “My anger prevented me from getting along with people as well as I’d have liked to”	1.40 (0.8)	0.90	1.90 (1.2)	0.92	1.25 (0.7)	.88	1.52 (1.0)	0.91
**DAR-3 Scale**								
Full Scale	4.97 (2.5)	0.90	6.53 (3.4)	0.92	5.04 (2.6)	.87	6.05 (3.2)	0.91
Individual items^b^								
1. “I found myself getting angry at people or situations”	2.01 (1.0)	0.90	2.54 (1.1)	0.92	1.86 (1.0)	.84	2.22 (1.2)	0.88
2. “When I got angry, I got really mad”	1.54 (0.9)	0.81	2.09 (1.3)	0.85	1.71 (1.0)	.78	2.06 (1.2)	0.84
3. “When I got angry, I stayed angry”	1.43 (0.9)	0.84	1.96 (1.2)	0.88	1.47 (0.9)	.83	1.76 (1.1)	0.87

Abbreviations: DAR-3, 3-item Dimensions of Anger Reactions; DAR-5, 5-item Dimensions of Anger Reactions.

^a^ In the US version, items were phrased slightly differently; see Adler et al.^1^

^b^ Individual item α are calculated as if that item were deleted.

### Concurrent Validity

Accuracy and Cohen κ were highest with a cutoff score of 8 of greater for 3 of 4 samples; additionally, this cutoff had an optimal degree of sensitivity and specificity across all 4 samples (Table 3). For the active duty US sample, a cutoff score of 8 or greater had a lower κ (κ = 0.86; 95% CI, 0.85-0.87) than a cutoff score of 9 or greater (κ = 0.87; 95% CI, 0.86-0.88), but the 8 or greater cutoff score had a higher sensitivity (0.95; 95% CI, 0.95-0.96 vs 0.83; 95% CI, 0.82-0.85). Considering these results, we determined that the higher sensitivity provided by a cutoff score of 8 or greater outweighed the marginal difference in Cohen κ scores.

**Table 3.** Positive Screening Results, Sensitivity, and Specificity of the DAR-3 in Australia and the US by Separation Status

Threshold	Estimate (95% CI)							
	Australia				US			
	Sensitivity	Specificity	Accuracy	κ	Sensitivity	Specificity	Accuracy	κ
7								
Active duty	0.99 (0.98-0.99)	0.92 (0.92-0.93)	0.93 (0.93-0.94)	0.75 (0.73-0.77)	0.99 (0.99-0.99)	0.91 (0.91-0.92)	0.92 (0.92-0.93)	0.72 (0.71-0.73)
Separated	0.99 (0.98-0.99)	0.88 (0.86-0.89)	0.92 (0.90-0.92)	0.82 (0.80-0.84)	0.99 (0.99-0.99)	0.88 (0.87-0.88)	0.91 (0.90-0.91)	0.77 (0.76-0.78)
8								
Active duty	0.94 (0.92-0.95)	0.98 (0.98-0.98)	0.97 (0.97-0.98)	0.89 (0.87-0.90)	0.95 (0.95-0.96)	0.97 (0.97-0.97)	0.97 (0.96-0.97)	0.86 (0.85-0.87)
Separated	0.95 (0.93-0.96)	0.96 (0.95-0.97)	0.96 (0.95-0.97)	0.91 (0.89-0.92)	0.96 (0.95-0.96)	0.96 (0.95-0.96)	0.96 (0.96-0.96)	0.89 (0.88-0.89)
9								
Active duty	0.81 (0.78-0.83)	1.00 (1.00-1.00)	0.97 (0.97-0.98)	0.87 (0.85-0.88)	0.83 (0.82-0.85)	0.99 (0.99-1.00)	0.97 (0.97-0.98)	0.87 (0.86-0.88)
Separated	0.84 (0.81-0.86)	0.99 (0.99-1.00)	0.94 (0.93-0.95)	0.86 (0.84-0.88)	0.85 (0.84-0.85)	0.99 (0.99-0.99)	0.96 (0.96-0.96)	0.88 (0.87-0.88)
10								
Active duty	0.55 (0.52-0.58)	1.00 (1.00-1.00)	0.94 (0.94-0.95)	0.68 (0.65-0.70)	0.61 (0.59-0.62)	1.00 (1.00-1.00)	0.95 (0.95-0.95)	0.73 (0.72-0.74)
Separated	0.64 (0.61-0.67)	1.00 (1.00-1.00)	0.88 (0.87-0.89)	0.70(0.67-0.73)	0.67 (0.66-0.68)	1.00 (1.00-1.00)	0.92 (0.92-0.92)	0.76 (0.75-0.77)

Abbreviation: DAR-3, 3-item Dimensions of Anger Reactions.

Using the cutoff of 8 or greater, the accuracy of DAR-3 in classifying problematic anger as defined on the DAR-5 (cutoff of ≥12) was robust in each sample (Table 4). Specifically, in the Australian sample, 7928 active duty participants (accuracy, 0.97; 95% CI, 0.97-0.98; κ = 0.89; 95% CI, 0.87-0.90), and 2639 separated participants (accuracy, 0.96; 95% CI, 0.95-0.97; κ = 0.91; 95% CI, 0.89-0.92) were accurately classified. In the US sample, 23 879 active duty participants (accuracy, 0.97; 95% CI, 0.96-0.97; κ = 0.86; 95% CI, 0.85-0.87) and 33 878 separated participants (accuracy, 0.96; 95% CI, 0.96-0.96; κ = 0.89; 95% CI, 0.88-0.89) were accurately classified. Combining all 4 samples, 68 324 participants (96.2%) were accurately classified. In relation to the DAR-5, the DAR-3 resulted in 2073 (2.9%) false positives and 613 (<1%) false negatives (Table 4).

**Table 4.** DAR-3 Confusion Matrix by Separation Status, Australia and the US

Finding	No. (%)	
	Australia	US
**Active duty**		
True positive	1004 (12.3)	2930 (11.9)
True negative	6924 (85.0)	20 949 (84.8)
False positive	150 (1.8)	687 (2.8)
False negative	67 (0.8)	140 (0.6)
**Separated**		
True positive	881 (32.0)	8090 (22.9)
True negative	1758 (63.8)	25 788 (72.8)
False positive	69 (2.5)	1167 (3.3)
False negative	47 (1.7)	359 (1.0)

Abbreviation: DAR-3, 3-item Dimensions of Anger Reactions.

### Comparison of the DAR-5 and DAR-3 Validity

In examining the bivariate associations of problematic anger with mental health and well-being, the ORs using the DAR-5 and DAR-3 were comparable (Table 5). Specifically, *z* tests comparing ORs from models estimating PTSD, depression, or relationship conflict from the DAR-5 and DAR-3 showed that the 2 scales were not significantly different across samples. Similarly, there were no significant differences in estimating aggression in the Australian samples; however, the magnitude of the estimate for the DAR-5 was significantly higher (active duty: OR, 9.96; 95% CI, 9.01-11.00; separated: OR, 12.36; 95% CI, 11.55-13.23) than the estimate using DAR-3 in the US samples (active duty: OR, 8.36; 95% CI, 7.58-9.22; *z* score, −1.96; *P* = .049; separated: OR, 10.59; 95% CI, 9.91-11.33; *z* score, −3.10; *P* = .002*)*.

**Table 5.** Associations of Problematic Anger, Comparing the DAR-3 and DAR-5, With Mental Health, Aggression, and Relationship Conflict

Measure	OR (95% CI)			
	PTSD	Depression	Aggression	Relationship conflict
**Australia**				
Active duty				
DAR-3	12.03 (10.40-13.93)	11.86 (10.28-13.69)	9.22 (5.89-14.64)	3.39 (2.91-3.95)
DAR-5	13.80 (11.87-16.08)	12.85 (11.10-14.88)	10.09 (6.44-16.02)	3.37 (2.88-3.96)
Separated				
DAR-3	16.91 (13.65-21.08)	15.02 (12.42-18.23)	12.52 (7.65-21.80)	3.63 (2.96-4.47)
DAR-5	19.35 (15.48-24.36)	14.83 (12.25-18.00)	13.01 (7.95-22.66)	3.82 (3.11-4.72)
**United States**				
Active duty				
DAR-3	13.73 (12.67-14.90)	13.69 (12.48-15.03)	8.36 (7.58-9.22)^a^	3.86 (3.59-4.16)
DAR-5	15.38 (14.10-16.77)	14.76 (13.43-16.22)	9.96 (9.01-11.00)^a^	4.18 (3.86-4.52)
Separated				
DAR-3	18.81 (17.69-20.00)	13.71 (12.93-14.53)	10.59 (9.91-11.33)^a^	4.35 (4.14-4.58)
DAR-5	21.39 (20.02-22.87)	14.02 (13.22-14.87)	12.36 (11.55-13.23)^a^	4.48 (4.25-4.72)

Abbreviations: DAR-3, 3-item Dimensions of Anger Reactions; DAR-5, 5-item Dimensions of Anger Reactions; OR, odds ratio; PTSD, posttraumatic stress disorder.

^a^ ORs were statistically significant in the models estimating aggression; however, estimates for estimating aggression were significantly lower when using the DAR-3 compared with the DAR-5 in the US active duty (*z* score, −1.96; *P* = .049) and separated (z score, −3.10; *P* = 0.002) samples.

### Supplemental Analyses and Materials

Overall, results for US Reserve and National Guard members were consistent with the primary models (eAppendix and eTable 1 in Supplement 1). DAR-3 items and response options are presented in eTable 2 in Supplement 1.

## Discussion

This cross-sectional study examined psychometric properties of a very brief measure of problematic anger using items from the DAR-5. Analyses were conducted with 2 large data sets of Australian and US military personnel, including active duty and separated service members. The 3 items selected from the DAR-5 to comprise the DAR-3 demonstrated strong psychometric properties, concurrent validity with the DAR-5, and validity with related constructs consistent with the DAR-5.

Much like the AUDIT-C selected items from the AUDIT that reflected alcohol consumption, the DAR-3 emphasizes the experience of anger. These 3 items, assessing anger frequency, intensity, and duration, represent the experiential component of anger without introducing possible consequences of that anger (eg, aggression and social functioning).

Analyses reinforced selection of these 3 items and provided empirical support for use of the DAR-3 as a briefer alternative to the DAR-5. First, the items demonstrated internal consistency, and each item contributed to the overall strength of the measure. Second, across all samples, the optimal DAR-3 cutoff score was 8 or greater. Third, using this cutoff, the DAR-3 was comparable with the DAR-5 in estimating key mental health variables (ie, PTSD, depression, and relationship conflict).

In contrast, models estimating aggression were not as consistent. In the Australian samples, the DAR-3 was comparable with the DAR-5 as a risk factor associated with aggression, despite the elimination of the 2 items relating to interpersonal conflict in the DAR-3. However, in the US samples, while the 2 measures estimated aggression, the DAR-3 was not as accurate as the DAR-5, although the difference in magnitude was small. The different patterns between the Australian and US samples may be due to the US aggression item capturing a broader range of behaviors than the Australian item. The more narrowly defined aggression item in the Australian survey may have contributed to their lower levels of endorsement, and in turn may have reduced power to detect differences between the DAR-5 and DAR-3 in the Australian samples. Nevertheless, the DAR-3 estimated aggression in all samples.

Given the prevalence of and risk associated with problematic anger in military and veteran populations, it is important to assess problematic anger in the military whenever possible. The DAR-3 offers a 40% reduction in survey items while still providing relevant information for researchers, program developers, and clinicians. The DAR-3 is well suited for population screening throughout the military and veteran lifecycle, and as a signal for intervention, whether through leaders, peers, clinicians, or self-management.

Indeed, the brevity and simplicity of the DAR-3 also makes it amenable for inclusion in online or mobile application–based self-management programs.^34^ By embedding the DAR-3 in such programs, it could be used to easily track self-reported change over time. The DAR-3 may also be helpful as a measure in intervention research. Moreover, as the need for brief, psychometrically sound measures of anger is not unique to the military context, there is value in conducting evaluations of the DAR-3 in nonmilitary populations.

### Limitations

This study has some limitations. Study limitations include the absence of a clinical interview to serve as a standard of comparison and the reliance on self-report. Moreover, nonresponse differed by age, race, and ethnicity; however, these differences were small, and prior investigations have found that nonresponse did not substantially change estimates of health outcomes.^35^ Furthermore, some measures differed between Australian and US samples. Additionally, results may not generalize to other countries or to nonmilitary populations; future research should consider addressing this gap.

## Conclusions

This cross-sectional study found that the DAR-3, with a cutoff score of 8 or greater, offered a reliable and valid approach to measuring problematic anger. These findings suggest that not only is this very brief measure suitable for assessing prevalence and signaling early risk, it could also be incorporated into intervention studies and self-management tools. The brevity of the DAR-3 could facilitate its inclusion in future research, advancing our understanding of problematic anger.

## Notes

Supplement 1. **eMethods.** Description of the Methods to Examine DAR-3 Among US National Guard and Reserve Service Members **eAppendix.** Findings From the US National Guard and Reserve Service Members **eTable 1.** Associations of Problematic Anger, Comparing DAR-3 and DAR-5, With Mental Health, Aggression, and Relationship Conflict Among US Reserve and National Guard Service Members **eTable 2.** 3-Item Dimensions of Anger Reactions Scale (DAR-3)

Supplement 2.
Data Sharing Statement

## References

[1] Adler AB, LeardMann CA, Roenfeldt KA, Jacobson IG, Forbes D; Millennium Cohort Study Team. Magnitude of problematic anger and its predictors in the Millennium Cohort. BMC Public Health. 2020;20(1):1168. doi:10.1186/s12889-020-09206-2 32718306 PMC7385895

[2] Varker T, Cowlishaw S, Baur J, . Problem anger in veterans and military personnel: prevalence, predictors, and associated harms of suicide and violence. J Psychiatr Res. 2022;151:57-64. doi:10.1016/j.jpsychires.2022.04.004 35453092

[3] Adler AB, LeardMann CA, Villalobos J, Jacobson IG, Forbes D; Millennium Cohort Study Team. Association of problematic anger with long-term adjustment following the military-to-civilian transition. JAMA Netw Open. 2022;5(7):e2223236. doi:10.1001/jamanetworkopen.2022.23236 35862043 PMC9305378

[4] Dell L, Casetta C, Benassi H, . Mental health across the early years in the military. Psychol Med. 2023;53(8):3683-3691. doi:10.1017/S0033291722000332 35197132 PMC10277765

[5] Wilks CR, Morland LA, Dillon KH, ; VA Mid-Atlantic MIRECC Workgroup. Anger, social support, and suicide risk in U.S. military veterans. J Psychiatr Res. 2019;109:139-144. doi:10.1016/j.jpsychires.2018.11.026 30537566

[6] Taft CT, Weatherill RP, Scott JP, Thomas SA, Kang HK, Eckhardt CI. Social information processing in anger expression and partner violence in returning U.S. veterans. J Trauma Stress. 2015;28(4):314-321. doi:10.1002/jts.22017 26201304

[7] Spielberger CD, Sydeman SJ, Owen AE, Marsh BJ. Measuring anxiety and anger with the State-Trait Anxiety Inventory (STAI) and the State-Trait Anger Expression Inventory (STAXI). In: Maruish ME, ed. The Use of Psychological Testing for Treatment Planning and Outcomes Assessment. 2nd ed. Lawrence Erlbaum Associates Publishers; 1999:993-1021.

[8] Forbes D, Alkemade N, Mitchell D, . Utility of the Dimensions of Anger Reactions–5 (DAR-5) scale as a brief anger measure. Depress Anxiety. 2014;31(2):166-173. doi:10.1002/da.22148 23801571

[9] Forbes D, Alkemade N, Hopcraft D, . Evaluation of the Dimensions of Anger Reactions–5 (DAR-5) scale in combat veterans with posttraumatic stress disorder. J Anxiety Disord. 2014;28(8):830-835. doi:10.1016/j.janxdis.2014.09.015 25445072

[10] Novaco R. Dimensions of Anger Reactions. University of California; 1975.

[11] Asmundson GJG, LeBouthillier DM, Parkerson HA, Horswill SC. Trauma-exposed community-dwelling women and men respond similarly to the DAR-5 anger scale: factor structure invariance and differential item functioning. J Trauma Stress. 2016;29(3):214-220. doi:10.1002/jts.22098 27166826

[12] Ceschi G, Selosse G, Nixon RDV, Metcalf O, Forbes D. Posttraumatic anger: a confirmatory factor analysis of the Dimensions of Anger Reactions Scale–5 (DAR-5)—French adaptation. Eur J Psychotraumatol. 2020;11(1):1731127. doi:10.1080/20008198.2020.1731127 32194923 PMC7067169

[13] Kakaje A, Alsamara K, Forbes D. Assessment of problematic anger using an Arabic adaptation of the Dimensions of Anger Reactions Scale–5 (DAR-5). J Affect Disord Rep. 2021;4:100128. doi:10.1016/j.jadr.2021.100128

[14] Ursano RJ, Colpe LJ, Heeringa SG, Kessler RC, Schoenbaum M, Stein MB; Army STARRS collaborators. The Army study to assess risk and resilience in servicemembers (Army STARRS). Psychiatry. 2014;77(2):107-119. doi:10.1521/psyc.2014.77.2.107 24865195 PMC4075436

[15] Zuromski KL, Ustun B, Hwang I, . Developing an optimal short-form of the PTSD Checklist for *DSM-5* (PCL-5). Depress Anxiety. 2019;36(9):790-800. doi:10.1002/da.22942 31356709 PMC6736721

[16] Morrison AS, Ustun B, Horenstein A, . Optimized short-forms of the Cognitive Distortions Questionnaire. J Anxiety Disord. 2022;92:102624. doi:10.1016/j.janxdis.2022.102624 36087565

[17] Reed-Fitzke K, LeardMann CA, Wojciak AS, . Identifying at-risk marines: a person-centered approach to adverse childhood experiences, mental health, and social support. J Affect Disord. 2023;325:721-731. doi:10.1016/j.jad.2023.01.020 36627058

[18] Bush K, Kivlahan DR, McDonell MB, Fihn SD, Bradley KA; Ambulatory Care Quality Improvement Project (ACQUIP). The AUDIT alcohol consumption questions (AUDIT-C): an effective brief screening test for problem drinking. Arch Intern Med. 1998;158(16):1789-1795. doi:10.1001/archinte.158.16.1789 9738608

[19] Kroenke K, Spitzer RL, Williams JBW. The Patient Health Questionnaire-2: validity of a two-item depression screener. Med Care. 2003;41(11):1284-1292. doi:10.1097/01.MLR.0000093487.78664.3C 14583691

[20] Van Hooff M, Lawrence-Wood E, Hodson S, Mental Health Prevalence, Mental Health and Wellbeing Transition Study. The Department of Defence and the Department of Veterans’ Affairs; 2018.

[21] Belding JN, Castañeda SF, Jacobson IG, ; Millennium Cohort Study Team. The Millennium Cohort Study: the first 20 years of research dedicated to understanding the long-term health of US Service Members and Veterans. Ann Epidemiol. 2022;67:61-72. doi:10.1016/j.annepidem.2021.12.002 34906635

[22] Smith TC; Millennium Cohort Study Team. The US Department of Defense Millennium Cohort Study: career span and beyond longitudinal follow-up. J Occup Environ Med. 2009;51(10):1193-1201. doi:10.1097/JOM.0b013e3181b73146 19786902

[23] Ryan MAK, Smith TC, Smith B, . Millennium Cohort: enrollment begins a 21-year contribution to understanding the impact of military service. J Clin Epidemiol. 2007;60(2):181-191. doi:10.1016/j.jclinepi.2006.05.009 17208125

[24] Terhakopian A, Sinaii N, Engel CC, Schnurr PP, Hoge CW. Estimating population prevalence of posttraumatic stress disorder: an example using the PTSD checklist. J Trauma Stress. 2008;21(3):290-300. doi:10.1002/jts.20341 18553416

[25] Kroenke K, Strine TW, Spitzer RL, Williams JBW, Berry JT, Mokdad AH. The PHQ-8 as a measure of current depression in the general population. J Affect Disord. 2009;114(1-3):163-173. doi:10.1016/j.jad.2008.06.026 18752852

[26] Shin C, Lee SH, Han KM, Yoon HK, Han C. Comparison of the usefulness of the PHQ-8 and PHQ-9 for screening for major depressive disorder: analysis of psychiatric outpatient data. Psychiatry Investig. 2019;16(4):300-305. doi:10.30773/pi.2019.02.01 31042692 PMC6504773

[27] McFarlane AC, Hodson SE, Van Hooff M, Davies C. Mental Health in the Australian Defence Force: 2010 ADF Mental Health and Wellbeing Study: Full Report. Department of Defence; 2011.

[28] Thomas JL, Wilk JE, Riviere LA, McGurk D, Castro CA, Hoge CW. Prevalence of mental health problems and functional impairment among active component and National Guard soldiers 3 and 12 months following combat in Iraq. Arch Gen Psychiatry. 2010;67(6):614-623. doi:10.1001/archgenpsychiatry.2010.54 20530011

[29] Brown JB, Lent B, Schmidt G, Sas G. Application of the Woman Abuse Screening Tool (WAST) and WAST-short in the family practice setting. J Fam Pract. 2000;49(10):896-903. 11052161

[30] Spitzer RL, Williams JB, Kroenke K, Hornyak R, McMurray J. Validity and utility of the PRIME-MD patient health questionnaire in assessment of 3000 obstetric-gynecologic patients: the PRIME-MD Patient Health Questionnaire Obstetrics-Gynecology Study. Am J Obstet Gynecol. 2000;183(3):759-769. doi:10.1067/mob.2000.106580 10992206

[31] Feuerman M, Miller AR. Relationships between statistical measures of agreement: sensitivity, specificity and kappa. J Eval Clin Pract. 2008;14(5):930-933. doi:10.1111/j.1365-2753.2008.00984.x 19018927

[32] Clogg CC, Petkova E, Haritou A. Statistical methods for comparing regression coefficients between models. Am J Sociol. 1995;100(5):1261-1293. doi:10.1086/230638

[33] Australian Government Department of Defence. Defence Census 2019: Public Report. Australian Government Department of Defence; 2020.

[34] Morland LA, Glassman LH, Mackintosh MA, Schnurr PP. Clinical interventions for problematic anger. In: Adler AB, Forbes D, eds. Anger at Work: Prevention, Intervention, and Treatment in High-Risk Occupations. American Psychological Association; 2021:247-273. doi:10.1037/0000244-009

[35] Littman AJ, Boyko EJ, Jacobson IG, ; Millennium Cohort Study. Assessing nonresponse bias at follow-up in a large prospective cohort of relatively young and mobile military service members. BMC Med Res Methodol. 2010;10(1):99. doi:10.1186/1471-2288-10-99 20964861 PMC2984503

