# Supplementary material for: Three-Item Dimensions of Anger Reactions Scale
Source: JAMA Netw Open. 2024 Feb 5;7(2):e2354741. doi: 10.1001/jamanetworkopen.2023.54741 (PMC10844994; doi:10.1001/jamanetworkopen.2023.54741)
Supplement: Supplement 1. — eMethods. Description of the Methods to Examine DAR-3 Among US National Guard and Reserve Service Members eAppendix. Findings From the US National Guard and Reserve Service Members eTable 1. Associations of Problematic Anger, Comparing DAR-3 and DAR-5, With Mental Health, Aggression, and Relationship Conflict Among US Reserve and National Guard Service Members eTable 2. 3-Item Dimensions of Anger Reactions Scale (DAR-3) [file jamanetwopen-e2354741-s001.pdf]

Supplemental Online Content

Forbes D, LeardMann CA, Lawrence-Wood E, et al. Three-item Dimensions of Anger Reactions scale. *JAMA Netw Open*. 2024;7(2):e2354741. doi:10.1001/jamanetworkopen.2023.54741

eMethods. Description of the Methods to Examine DAR-3 Among US National Guard and Reserve Service Members

eAppendix. Findings From the US National Guard and Reserve Service Members

eTable 1. Associations of Problematic Anger, Comparing DAR-3 and DAR-5, With Mental Health, Aggression, and Relationship Conflict Among US Reserve and National Guard Service Members.

eTable 2. 3-Item Dimensions of Anger Reactions Scale (DAR-3)

This supplemental material has been provided by the authors to give readers additional information about their work.

© 2024 Forbes D et al. *JAMA Network Open.*

eMethods

A supplemental analysis was conducted to examine the relative psychometric properties of the 3-item Dimensions of Anger Reactions (DAR-3) scale in a sample of US Reserve and National Guard (R/NG) members. This sample consisted of current and former US R/NG service members who completed the 2014-2016 Millennium Cohort survey, and who had not previously served as regular active duty service members (n=40 244). Like in the main paper, these R/NG supplemental analyses were stratified by military status (current: n=16 750; separated: n=23 474). Participants missing 1 or more DAR items were excluded from these analyses (current: n=779; separated: n=747), resulting in a total of 15 971 current and 22 727 separated R/NG members.

eAppendix.

Overall, the results for these US R/NG samples were consistent with the results from the US samples in the main analyses. For both the current and separated R/NG samples, the DAR-5 (current: α =0.90; separated: α =0.92) and DAR-3 (current: α = 0.88; separated: α =0.90) had good internal consistency. When each of the first 3 DAR-5 items was deleted, internal consistency decreased slightly (current: 0.87-.88; separated: 0.89-0.90); internal consistency decreased less for the final 2 DAR-5 items (i.e., aggressive impulses and interference with social functioning), which had the same α if item deleted (current: 0.89; separated: 0.91). For the current R/NG members, Cohen's κ for the DAR-3 with a cut-off score of 8 (0.86, 95% CI: 0.85, 0.87) was nominally lower than the DAR-3 cut-off score of 9 (0.88, 95% CI: 0.87, 0.89), but associated with higher sensitivity (0.95, 95% CI: 0.94, 0.96 and 0.83, 95% CI: 0.82, 0.85, respectively). For the separated R/NG members, Cohen's κ for the DAR-3 with a cut-off score of 8 (0.88, 95% CI: 0.87, 0.89) was the same as the DAR-3 cut-off score of 9 (0.88, 95% CI: 0.87, 0.88), and associated with higher sensitivity (0.96, 95% CI: 0.95, 0.96 and 0.84, 95% CI: 0.82, 0.85, respectively). Consistent with the main analyses, a cut-off score of 8 was determined to be optimal since it was associated with high sensitivity and outweighed the marginal difference in Cohen's κ scores. Based on these respective cut-off scores at least 12 for the DAR-5 and at least 8 for the DAR-3), 1995 (12.5%) and 2298 (14.4%) of the current R/NG members and 4106 (18.1%) and 4604 (20.3%) of the separated R/NG members screened positive for problematic anger using the DAR-5 and DAR-3, respectively. In relation to the DAR-5, the DAR-3 produced 409 (2.6%) and 670 (2.9%) false positives and 106 (0.7%) and 172 (0.8%) false negatives for the current and separated R/NG samples, respectively. In examining the association of mental health and well-being with problematic anger, the odds ratios using the DAR-5 and DAR-3 were comparable (eTable 1). The z-tests comparing the odds ratios from models predicting PTSD, depression, or relationship conflict from the DAR-5 and DAR-3 showed that the two scales were not significantly different. However, the DAR-5 was more predictive of aggression for separated R/NG members. This overall pattern was consistent with the US samples in the main analyses. Thus, results from these supplemental analyses suggest that the DAR-3, with a cut off score of 8 or more, is a reliable and valid streamlined measure of problematic anger for current and separated R/NG members.

eTable 1. Associations of problematic anger, comparing DAR-3 and DAR-5, with mental health, aggression, and relationship conflict among current and separated US Reserve and National Guard members.

	PTSD	Depression	Aggression	Relationship conflict
	OR (95% CI)	OR (95% CI)	OR (95% CI)	OR (95% CI)
Current Reserve/National Guard members				
DAR-3	18.23 (16.42, 20.27)	16.36 (14.58, 18.37)	9.96 (8.88, 11.18)	4.96 (4.52, 5.46)
DAR-5	21.60 (19.26, 24.26)	18.07 (16.07, 20.32)	12.21 (10.86, 13.73)	5.13 (4.64, 5.68)
Separated Reserve/National Guard members				
DAR-3	20.08 (18.50, 21.81)	16.35 (15.08, 17.74)	11.23[a] (10.29, 12.26)	5.13 (4.78, 5.50)
DAR-5	24.43 (22.32, 26.78)	17.91 (16.49, 19.46)	13.14[a] (12.03, 14.36)	5.30 (4.92, 5.71)

[a]ORs were statistically significant in the individual models predicting aggression for both samples; estimates for predicting aggression were significantly lower when using the DAR-3 compared with the DAR-5 (z-score: -2.36, p-value=0.02).

© 2024 Forbes D et al. *JAMA Network Open.*

eTable 2. 3-item Dimensions of Anger Reactions scale (DAR-3)

Thinking **over the past 4 weeks**, circle the number under the option that best describes the amount of time you felt that way.

		None or almost none of the time	A little of the time	Some of the time	Most of the time	All or almost all of the time
1	I found myself getting angry at people or situations	1	2	3	4	5
2	When I got angry, I got really mad	1	2	3	4	5
3	When I got angry, I stayed angry	1	2	3	4	5

Scoring instructions: Sum responses to the 3 items (range: 3-15); a score of ≥8 indicates problematic anger.
