# Supplementary material for: Three-Item Dimensions of Anger Reactions Scale
Source: JAMA Netw Open. 2024 Feb 5;7(2):e2354741. doi: 10.1001/jamanetworkopen.2023.54741 (PMC10844994; doi:10.1001/jamanetworkopen.2023.54741)
Supplement: Supplement 2. — Data Sharing Statement [file jamanetwopen-e2354741-s002.pdf]

Data Sharing Statement

Forbes. Three-Item Dimensions of Anger Reactions Scale. *JAMA Netw Open*. Published February 05, 2024. doi:10.1001/jamanetworkopen.2023.54741

Data
Data available: No
